# Estimate of the prevalence of depression among older people in Africa: a systematic review and meta-analysis

**DOI:** 10.1192/j.eurpsy.2022.1665

**Published:** 2022-09-01

**Authors:** A. Tilahune, N. Mekonnen, B. Duko

**Affiliations:** 1 University of Technology Sydney, Public Health, Sydney, Australia; 2 Hawassa University, Nursing, Hawassa, Ethiopia; 3 Curtin University, Public Health, Perth, Australia

**Keywords:** Elderly, Africa, Prevalence, Depression

## Abstract

**Introduction:**

Among non-communicable diseases, depression is a leading cause of morbidity in elderly people with varying magnitude across countries. No systematic review and meta-analysis has yet examined the pooled prevalence of depression among elderly in Africa.

**Objectives:**

The current systematic review and meta-analysis aimed to estimate the pooled prevalence of depression among elderly people in Africa.

**Methods:**

We have searched CINAHL, PubMed, SCOPUS and Psych-iNFO databases to identify observational studies which reported the prevalence of depression among the elderly. We used a random-effects model due to reported heterogeneity among the studies. The publication bias was examined by using egger’s test, visual inspection of the symmetry in funnel plots and adjusted using Trim and Fill analysis. We used Cochran’s Q and the I2-tests to measure heterogeneity across the studies.

**Results:**

A total of 23 studies conducted in Africa were included in the current systematic review and meta-analysis, representing a total of 14, 350 elderly population. The pooled prevalence of depression among elderly people in Africa was estimated to be 26.3% (95% Ci; 22.2, 30.4%). The estimated pooled prevalence of depression among the elderly in Africa was much higher (43.1%) in studies that used a screening tool to measure depression when compared to studies that used a diagnostic tool (24.2%). Also, the prevalence of depression among female elderly participant (43.10%) was higher than that of male elderly participant (30.90%).

**Conclusions:**

One in five elderly population in Africa were depressed. Timely and targeted screening of depression among the elderly and evidence-based interventions were highly recommended.

**Disclosure:**

No significant relationships.

